# Regional inequalities in vaccination coverage and COVID-19 risk among children and adolescents: spatial analysis, Brazil, 2020–2024

**DOI:** 10.1590/S2237-962220v35e20251042.en

**Published:** 2026-07-24

**Authors:** Reginaldo Bazon Vaz Tavares, Yan Mathias Alves, Nathalia Zini, Ariela Fehr Tártaro, Maria Eduarda Pagano Pelodan, Luanne Karolyne Leal dos Santos, Daniele de Moraes Pinheiro, Tatiana Ferraz de Araújo Alecrim, Ricardo Alexandre Arcêncio

**Affiliations:** 1 Universidade de São Paulo Escola de Enfermagem de Ribeirão Preto Ribeirão Preto SP Brazil Universidade de São Paulo, Escola de Enfermagem de Ribeirão Preto, Ribeirão Preto, SP, Brazil

**Keywords:** COVID-19, Child, Vaccines, Risk, Spatial Analysis, COVID-19, Niño, Vacunas, Riesgo, Análisis Espacial

## Abstract

**Objective:**

To identify space-time risk clusters for cases and deaths from severe acute respiratory syndrome (SARS) caused by COVID-19 and to characterize the spatial distribution of vaccination coverage among children and adolescents aged 0–19 years in Brazil.

**Methods:**

Spatial analysis study using secondary data from the 5,570 municipalities in Brazil. Cases and deaths from SARS caused by COVID-19 among children and adolescents aged 0–19 years reported between March 2020 and December 2024 in the Influenza Epidemiological Surveillance Information System (*Sistema de Informação da Vigilância Epidemiológica da Gripe*, SIVEP-Gripe) were included. Vaccination records from January 2021 to December 2024 were analyzed. Vaccination coverage rates were calculated by age group and vaccination schedule. Space-time clusters were identified using the spatial scan technique.

**Results:**

A total of 74,470 cases and 3,749 deaths were recorded in the population aged 0–19 years. Significant clusters of high and low risk were identified, including one protective cluster for cases among individuals aged 0–19 years (relative risk [RR] 0.28; 95% confidence interval [95%CI] 0.27; 0.29) and another high-risk cluster for deaths in the North, Northeast, and Central-West regions (RR 3.28; 95%CI 3.00; 3.59). Primary vaccination reached high coverage (85.2%), especially among adolescents aged 15–19 years (96.8%), whereas booster dose coverage remained insufficient (23.1%). The spatial distribution showed heterogeneity, with lower vaccination coverage in the North and Central-West regions.

**Conclusion:**

Space-time inequalities were observed in cases and deaths from severe acute respiratory syndrome (SARS) caused by COVID-19 among children and adolescents, with risk clusters and heterogeneity in vaccination coverage, characterized by high primary coverage and low booster coverage, especially in the North and Central-West regions..

## Resumen

**Objetivo:**

Identificar conglomerados espaciotemporales de riesgo para casos y muertes por síndrome respiratorio agudo grave (SARS) causado por covid-19 y caracterizar la distribución espacial de la cobertura de vacunación entre niños y adolescentes de 0 a 19 años en Brasil.

**Métodos:**

Estudio de análisis espacial con datos secundarios de los 5.570 municipios de Brasil. Se consideraron casos y muertes por SARS causados por covid-19 en niños y adolescentes de 0 a 19 años, notificados entre marzo de 2020 y diciembre de 2024, en el Sistema de Información de la Vigilancia Epidemiológica de la Gripe (Sistema de Informação de Vigilância Epidemiológica da Gripe, SIVEP-Gripe). Se analizaron registros de vacunación entre enero de 2021 y diciembre de 2024. Las tasas de cobertura vacunal se calcularon por grupo de edad y por esquema de vacunación. Los conglomerados espaciotemporales se identificaron mediante la técnica de escaneo espacial.

**Resultados:**

Se registraron 74.470 casos y 3.749 muertes en la población de 0 a 19 años. Se identificaron conglomerados significativos de alto y de bajo riesgo, incluido uno de protección para casos en personas de 0 a 19 años (riesgo relativo [RR] 0,28; intervalos de confianza del 95% [IC95%] 0,27; 0,29) y otro de alto riesgo para muertes en las regiones Norte, Nordeste y Centro-Oeste (RR 3,28; IC95% 3,00; 3,59). La vacunación primaria alcanzó una alta cobertura (85,2%), especialmente entre adolescentes de 15-19 años (96,8%), mientras que la dosis de refuerzo permaneció insuficiente (23,1%). La distribución espacial evidenció heterogeneidad, con coberturas de vacunación menores en el Norte y en el Centro-Oeste.

**Conclusión:**

Se evidenciaron desigualdades espaciotemporales en los casos y en las muertes por SARS de covid-19 entre niños y adolescentes, con conglomerados de riesgo y heterogeneidad en la cobertura vacunal, marcada por alta cobertura primaria y bajo refuerzo, especialmente en el Norte y el Centro-Oeste.

**Ethical aspects**

This research used public domain anonymized databases.

## Introduction

The COVID-19 pandemic, caused by the SARS-CoV-2 virus, imposed major challenges on health systems worldwide [1]. Brazil was one of the countries most severely affected by this public health emergency, with a total of 39,295,043 cases and 716,509 deaths by August 2025 [2]. Since the beginning of the pandemic, the disease has been known to present greater severity in adults and older adults, particularly among those with comorbidities [3].

Among children and adolescents, COVID-19 tends to be less severe, with mild clinical presentations and lower case fatality than in adults [3,4,5]. However, hospitalizations, deaths, and complications such as multisystem inflammatory syndrome in children were frequent, especially in contexts of greater vulnerability, in low- and middle-income countries, among individuals with comorbidities, and in the first years of life [4].

Brazil has already been the country with the highest COVID-19 mortality rate among children and adolescents, with 23.6 deaths per 1,000,000 children recorded in 2020 [4]. This highlights the need for more effective and equitable strategies to protect this group. The progressive inclusion of children and adolescents as a priority group in COVID-19 vaccination campaigns, followed by their incorporation into the routine immunization schedule, represented an important milestone in addressing the pandemic, as it expanded collective protection and contributed to equity in health actions.

COVID-19 vaccination coverage among children and adolescents (0–19 years) remains low in several countries, which helps explain its global public health priority status, aligned with the United Nations 2030 Agenda, especially Sustainable Development Goal 3 (Good Health and Well-being) [6]. In Australia, by September 2022, 51.3% of children had received the first dose and 40.4% the second; in the United States, by February 2023, these proportions were 39.0% and 32.0%, respectively; and in Canada, by 2023, 67.1% of the population younger than 18 years had received at least one vaccine dose [7,8]. These insufficient levels reveal strong regional and social heterogeneity, associated with socioeconomic inequalities and unequal access to health services [8]. In Brazil, marked disparities are observed, with higher coverage in the South and Southeast and lower coverage in the North and Northeast, related to social, geographic, and logistical factors [8].

Although COVID-19 is less severe among children and adolescents compared with adults, mortality in this population remains significant, especially in socioeconomically disadvantaged contexts [9,10,11,12,13]. It should be noted that inequalities in vaccination coverage may influence geographic patterns of risk and preventable deaths [3,5]. However, after the acute phase of the pandemic, no study has sought to analyze this phenomenon using integrated analyses in Brazil.

This study analyzes, from a space-time perspective, morbidity and mortality and COVID-19 vaccination coverage among children and adolescents in Brazil, seeking to fill gaps in the literature and to support equitable public policies aligned with the Sustainable Development Goals. The objective of the study was to identify space-time clusters of risk for cases and deaths from severe acute respiratory syndrome (SARS) caused by COVID-19 and to characterize the spatial distribution of COVID-19 vaccination coverage among children and adolescents aged 0–19 years in Brazil.

## Methods

### Study design

This is a spatial analysis study using secondary population-based data from Brazil’s 5,570 municipalities.

### Setting

Brazil, the largest country in Latin America, has a vast territorial extension of 8,509,379,576 km^2^ and an estimated population of 213,421,037 inhabitants. Organized into 26 states and the Federal District, the country is divided into five regions: North, Northeast, Central-West, South, and Southeast [14]. This geographic dimension reflects a diversity of social, political, and economic processes that, in turn, perpetuate profound regional inequality, especially in income distribution and access to essential services such as health and education [15].

### Participants

All reported cases and deaths from SARS caused by COVID-19 among children and adolescents aged 0–19 years in Brazil between March 2020 and December 2024 were included. All included cases were confirmed by laboratory testing, according to the criteria adopted by the Ministry of Health [16].

For COVID-19 vaccination data, records of administered doses between January 2021 and December 2024 were used, considering all types of vaccines available and offered during this period.

### Data sources and measurement

Data on SARS caused by COVID-19 were obtained from the Influenza Epidemiological Surveillance Information System (*Sistema de Informação da Vigilância Epidemiológica da Gripe*, SIVEP-Gripe), a system implemented in 2000 to monitor respiratory viruses circulating in Brazil [17].

Data on COVID-19 vaccination were obtained from the Ministry of Health open data portal, through the Department of Information and Informatics of the Brazilian Unified Health System (*Departamento de Informação e Informática do Sistema Único de Saúde*, DATASUS), extracted in April 2025.

Population data by age group were obtained from the 2022 demographic census, available on the Brazilian Institute of Geography and Statistics (*Instituto Brasileiro de Geografia e Estatística*, IBGE) portal.

### Variables

For this study, the number of reported SARS cases caused by COVID-19 was used. Cases were filtered by etiologic agent classification, considering only those confirmed by laboratory criteria that detected SARS-CoV-2. Age for aggregation of age groups was defined based on the “age” field reported in each notification. Records in which inconsistent values or missing data were identified were excluded.

To account for deaths from COVID-19, the case outcome classification for SARS caused by COVID-19 was used, considering cases whose treatment outcome was recorded as death and following the same selection criteria described above.

Both datasets were stratified by age groups, namely: 0–4 years, 5–9 years, 10–14 years, 15–19 years, and 0–19 years. This definition was based on the standardization of age-group–specific rates recommended by the World Health Organization [18] to enable comparability of results across different contexts.

Vaccination coverage rates were calculated at the municipal and national levels using records of administered COVID-19 vaccine doses. Three categories of coverage were considered: (i) primary vaccination schedule (single dose and second dose), (ii) booster dose, and (iii) third dose. Based on this, coverage rates were calculated according to the following formula:


$$\mathrm{Vaccination}\;\mathrm{coverage}=\frac{\mathrm{number}\;\mathrm{of}\;\mathrm{doses}\;\mathrm{administered}\;(\mathrm{according}\;\mathrm{to}\;\mathrm{age}\;\mathrm{group}\;\mathrm{and}\;\mathrm{vaccination}\;\mathrm{category})\;}{\mathrm{population}\;\mathrm{by}\;\mathrm{age}\;\mathrm{group}}*100$$

COVID-19 vaccination began in Brazil on 17 January 2021. Vaccination of children and adolescents began on June 11, 2021, following the approval of the Pfizer vaccine for administration in adolescents aged 12–17 years. In December of the same year, vaccination began for children aged 5–11 years with the same vaccine [19].

In July 2022, children aged 3–5 years became eligible for vaccination with CoronaVac. In September 2022, the Ministry of Health approved the pediatric Pfizer vaccine for children aged 6 months to under 5 years. From November 2022 onward, vaccination with the bivalent vaccine was recommended for individuals aged 12 years and older with comorbidities and/or immunosuppression [19].

For the spatial analysis, the municipality of residence was used to determine the municipality of occurrence of each case, death, or vaccination record. The date of notification was used as the temporal reference for cases and deaths, and the date of vaccine administration as the reference for vaccination records.

### Statistical analysis methods

### Cluster detection

To detect space-time clusters of cases and deaths from COVID-19 among children and adolescents, the Scan statistics technique [20,21] was used, with the 5,570 Brazilian municipalities as the unit of analysis. In this technique, information about the event’s occurrence is associated with a single point on the map, defined for each unit of analysis studied and called the centroid. From this, the entire study area is examined by creating cylinders of variable dimensions around the centroids.

Clusters are identified by calculating the expected and observed numbers of events. When the number of expected events differs significantly from the number of observed events (either higher or lower), a cluster is defined. If this difference is not significant, the base (spatial dimension) or the height (temporal dimension) of the cylinder is expanded until all centroids, and the entire study period have been tested [22,23].

The statistical test considers the null hypothesis (H₀) to be the absence of clusters in the study region, and the alternative hypothesis (H₁) to be that a given region Z constitutes a cluster. For the identified clusters, the relative risk (RR) is calculated, which determines whether the cluster represents an area of risk (>1) or protection (<1) [22,23].

A retrospective approach was adopted, and since these are count data (number of cases and deaths), the Poisson model was used. The analysis parameters included: no geographic overlap of clusters; clusters with a maximum size of 50% of the exposed population and 50% of the study period; circular cluster shape; 999 Monte Carlo replications; and temporal precision in months, for the period from March 2020 to December 2024. Clusters with p-value<0.05 were considered statistically significant. The analysis was performed using SaTScan software version 10.0.2 (https://www.satscan.org/).

For spatial visualization of the results, choropleth maps were constructed to show the identified clusters and their respective RR values and 95% confidence intervals (95%CI), using ArcGIS software version 10.5 (https://www.esri.com/). Observed and expected case and death counts for each cluster were also presented.

### Time series

Monthly time series of cases and deaths were developed for each age group, accounting for event occurrence within the identified clusters. Analyses were conducted using R (version 4.2.1) via RStudio (version 2025.05.1).

### Spatial distribution of vaccination coverage

To map COVID-19 vaccination coverage, choropleth maps were constructed using municipalities as the unit of analysis, stratified by age group and vaccination schedule type. The maps were produced in R using RStudio (version 2025.05.1).

## Results

A total of 74,470 cases of SARS caused by COVID-19 and 3,749 deaths were recorded among children and adolescents aged 0–19 years between March 2020 and December 2024.

Table 1 presents the annual incidence and mortality rates for SARS by age group. A higher incidence was observed among children aged 0–4 years (71.7 cases per 100,000 inhabitants), followed by adolescents aged 15–19 years (17.7 cases per 100,000 inhabitants) and children aged 5–9 years (15.9 cases per 100,000 inhabitants).

**Table 1:** Incidence and mortality rates of severe acute respiratory syndrome caused by COVID-19 among children and adolescents aged 0–19 years, by age group. Brazil, 2020–2024 (n=74,470)

Age group (years)	Annual incidence rate (cases per 100,000 inhabitants)	Annual mortality rate (deaths per 100,000 inhabitants)
0–19	27.3	1.4
0–4	69.4	3.0
5–9	15.4	0.5
10–14	10.8	0.6
15–19	17.1	1.5

The highest mortality rates were also recorded in the 0–4-year age group, with 3.1 deaths per 100,000 inhabitants, followed by adolescents aged 15–19 years (1.5 deaths per 100,000). The lowest mortality rates were observed among children aged 5–9 years (0.5 deaths per 100,000) and in the 10–14-year age group (0.6 deaths per 100,000).

The space-time scan analysis identified statistically significant clusters of high risk and protection for cases (Figure 1) and deaths (Figure 2), considering all age groups studied.

**Figure 1:**
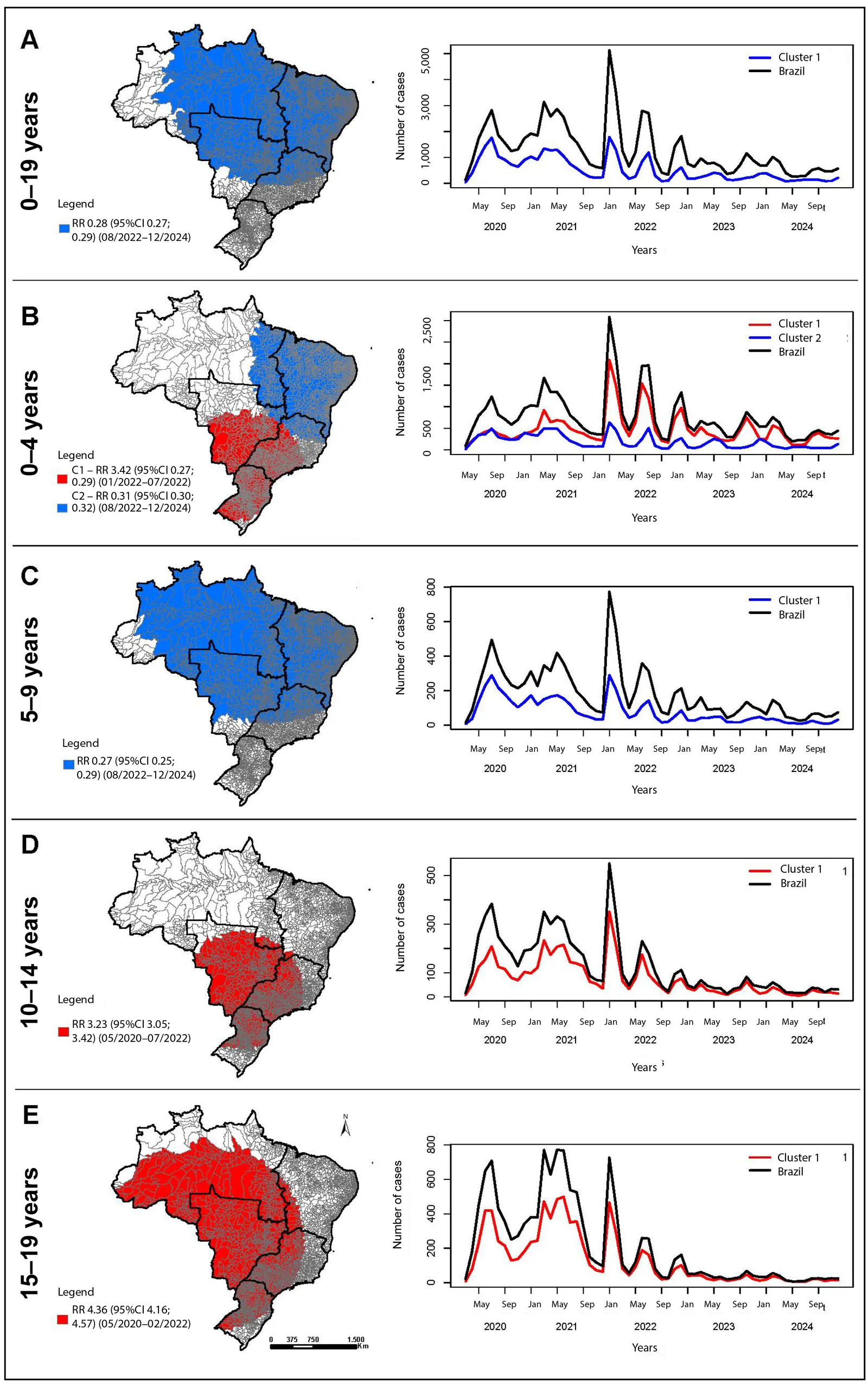
Space-time clusters and time series of reported severe acute respiratory syndrome deaths caused by COVID-19 in children and adolescents aged (A) 0–19 years, (B) 0–4 years, (C) 5–9 years, (D) 10–14 years, and (E) 15–19 years. Brazil, 2020–2024 (n=74,470)

**Figure 2:**
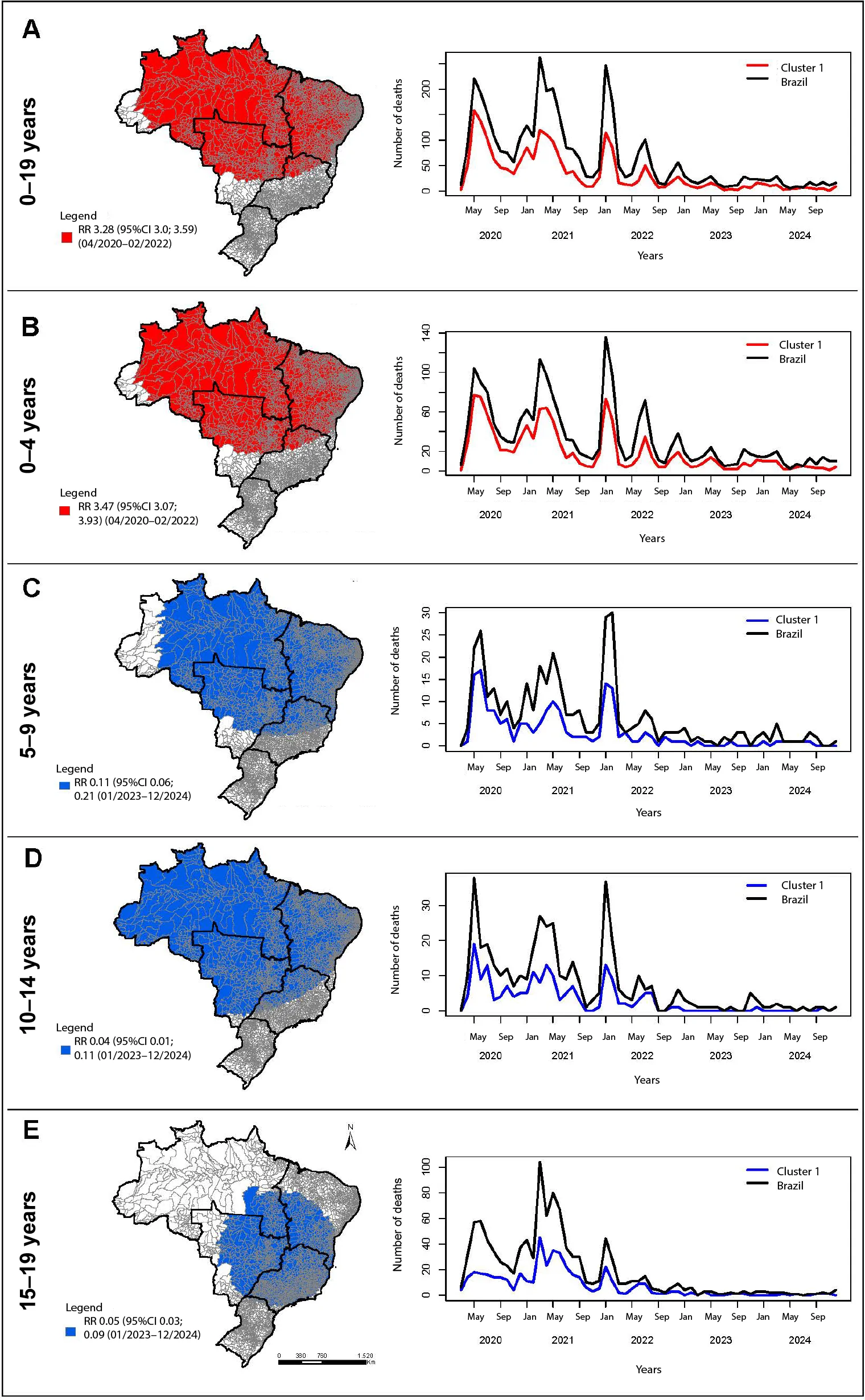
Space-time clusters and time series of reported severe acute respiratory syndrome deaths caused by COVID-19 in children and adolescents aged (A) 0–19 years, (B) 0–4 years, (C) 5–9 years, (D) 10–14 years, and (E) 15–19 years. Brazil, 2020-2024 (n=3,749)

In the 0–19-year age group (Figure 1A), one cluster was detected with an RR of 0.28 (95%CI 0.27; 0.29), comprising a population of 27,242,116 people, with 6,444 observed cases and 18,592.36 expected cases, mainly in the North, Northeast, and Central-West regions.

In the 0–4-year age group (Figure 1B), two clusters were identified. Cluster 1 had an RR of 3.42 (95%CI 3.27; 3.57), comprising a population of 6,334,196 people, with 7,885 observed cases and 2,637.79 expected cases, mainly in the South region and parts of the Central-West and Southeast regions. Cluster 2 had an RR of 0.31 (95%CI 0.30; 0.32), comprising a population of 4,509,684 people, with 2,800 observed cases and 7,830.88 expected cases, mainly in the Northeast region and parts of the Southeast and North regions.

In the 5–9-year age group (Figure 1C), one cluster was identified with an RR of 0.27 (95%CI 0.25; 0.29), comprising a population of 6,869,811 people, with 870 observed cases and 2,641.86 expected cases, covering the entire Northeast region and parts of the Central-West, Southeast, and North regions.

In the 10–14-year age group (Figure 1D), one cluster was identified with an RR of 3.23 (95%CI 3.05; 3.42), comprising a population of 6,660,105 people, with 3,596 observed cases and 1,675.66 expected cases, covering the South, Southeast, and Central-West regions.

In the 15–19-year age group (Figure 1E), one cluster was identified with an RR of 4.36 (95%CI 4.16; 4.57), comprising a population of 7,174,512 people, with 6,195 observed cases and 214.05 expected cases, covering the entire Central-West region and parts of the North and Southeast regions.

Regarding deaths from COVID-19, one cluster was detected with an RR of 3.28 (95%CI 3.00; 3.59) in the 0–19-year age group (Figure 2A), comprising a population of 25,563,551 people, with 1,602 observed deaths and 694.45 expected deaths, in the North, Northeast, and Central-West regions.

In the 0–4-year age group (Figure 2B), one cluster was identified with an RR of 3.47 (95%CI 3.07; 3.93), comprising a population of 5,975,208 people, with 846 observed deaths and 356.09 expected deaths, in the North, Northeast, and Central-West regions.

In the 5–9-year age group (Figure 2C), one cluster was identified with an RR of 0.11 (95%CI 0.06; 0.21), comprising a population of 6,874,405 people, with 10 observed deaths and 75.5 expected deaths, in the North, Northeast, and Central-West regions.

One cluster was identified in the 10–14-year age group (Figure 2D), with an RR of 0.04 (95%CI 0.01; 0.11), comprising a population of 6,607,633 people, with 4 observed deaths and 83.76 expected deaths, covering the entire North, Northeast, and Central-West regions.

In the 15–19-year age group (Figure 2E), one cluster was identified with an RR of 0.05 (95%CI 0.03; 0.09), comprising a population of 7,117,967 people, with 14 observed deaths and 214.05 expected deaths, mainly covering the Southeast region and parts of the North, Northeast, and Central-West regions.

Table 2 shows high coverage of the primary COVID-19 vaccination schedule among children and adolescents, especially among those aged 15–19 years (96.8%). Booster uptake, however, was heterogeneous, ranging from 0.4% (0–4 years) to 49.7% (15–19 years). Third-dose coverage was low across all age groups, indicating challenges in expanding vaccination.

**Table 2:** COVID-19 vaccination coverage among the population aged 0–19 years in Brazil, by age group and vaccination schedule. Brazil, 2020–2024 (n=120,607,559)

Vaccination schedule/age group (years)	Total doses	Population	Coverage (%)
**Primary vaccination**			
0–19	46,421,022	54,505,203	85.2
0–4	7,964,519	12,704,860	62.7
5–9	11,575,968	13,749,440	84.2
10–14	11,575,968	13,749,440	84.2
15–19	13,918,636	14,375,942	96.9
**Booster dose**			
0–19	12,572,858	54,505,203	23.1
0–4	45,484	12,704,860	0.4
5–9	1,465,249	13,749,440	10.7
10–14	3,915,185	13,674,961	28.6
15–19	7,146,940	14,375,942	49.7
**Third dose**			
0–19	2,002,865	54,505,203	3.7
0–4	1,062,030	12,704,860	8.4
5–9	440,765	13,749,440	3.2
10–14	322,401	13,674,961	2.4
15–19	177,669	14,375,942	1.2

Figure 3 presents the spatial distribution of COVID-19 vaccination coverage among children and adolescents in Brazil, stratified by age group and vaccination schedule (primary schedule, first booster dose, and third booster dose).

**Figure 3:**
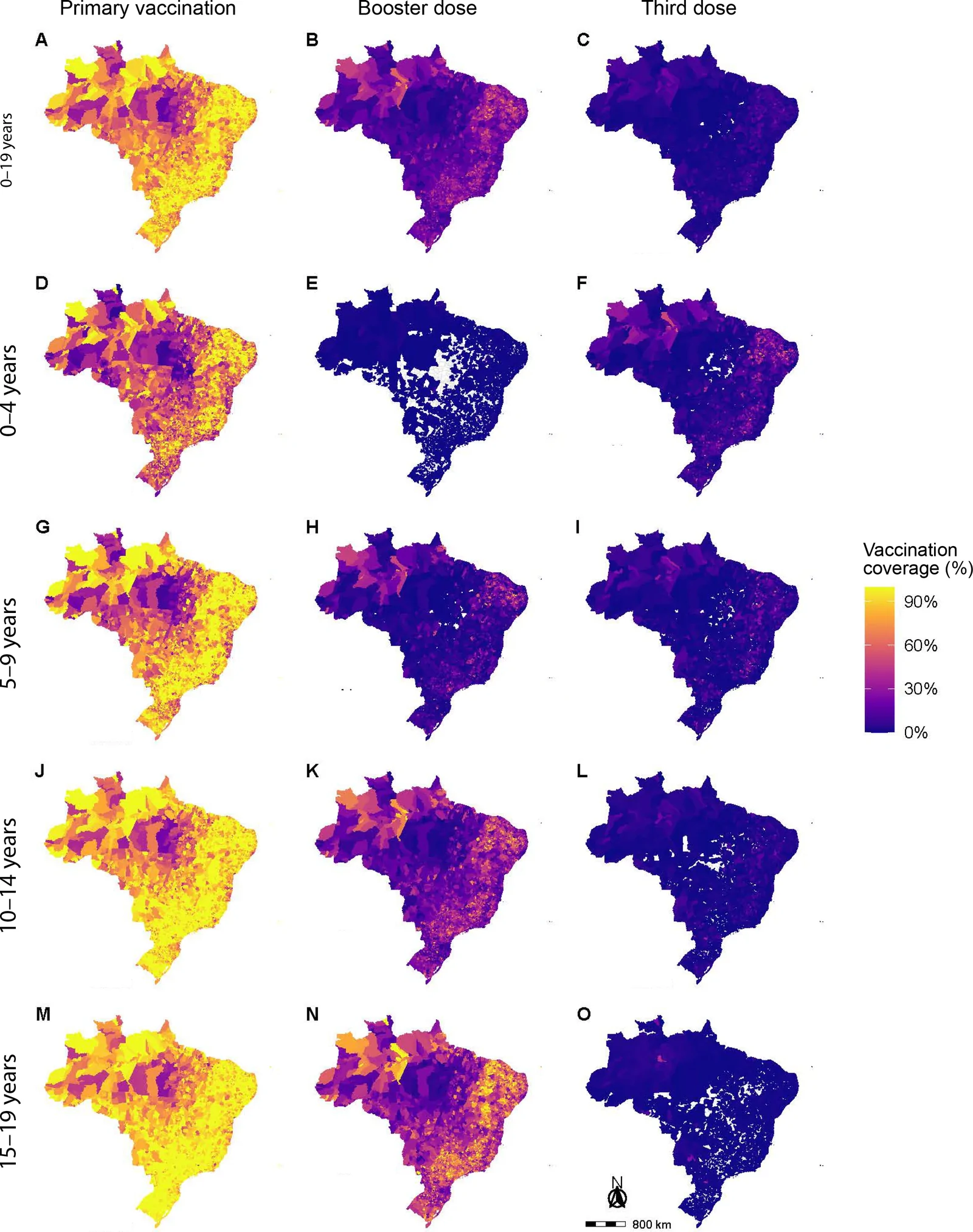
Spatial distribution of COVID-19 vaccination coverage among children and adolescents, by age group, for the primary vaccination: (A) 0–19 years, (D) 0–4 years, (G) 5–9 years, (J) 10–14 years, (M) 15–19 years; for the booster dose: (B) 0–19 years, (E) 0–4 years, (H) 5–9 years, (K) 10–14 years, (N) 15–19 years; and for the third dose: (C) 0–19 years, (F) 0–4 years, (I) 5–9 years, (L) 10–14 years, (O) 15–19 years. Brazil, 2020–2024 (n=120,607,559)

There was high coverage of the primary schedule, particularly from age 5 onward, with higher percentages in the South, Southeast, and Northeast regions (Figures 3G, 3J, 3M). The North and Central-West regions showed pockets of low coverage, especially among children younger than 4 years (Figure 3D). Booster dose coverage (Figure 3E) was more heterogeneous, with low coverage in younger age groups. In contrast, the third dose showed low uptake across all ages (Figures 3F, 3I, 3L, 3O), with marked territorial inequality and lower coverage in the Central-West region.

## Discussion

The findings indicated that the occurrence of COVID-19 cases and deaths among children and adolescents in Brazil showed marked territorial heterogeneity. The identification of areas of higher risk and protection indicates that the distribution of the disease was not homogeneous across municipalities and regions, reflecting the influence of distinct epidemiological and social contexts. These results reinforce the relevance of a space-time approach to understanding the dynamics of COVID-19 in this age group and to support surveillance and intervention strategies in specific territories.

This study has some limitations. The use of SIVEP-Gripe limited the analysis to severe cases of COVID-19, thereby excluding mild or asymptomatic infections, which are more frequent among children and adolescents. The effects of underreporting and reporting bias should be considered, particularly in socially vulnerable areas, which may result in underestimation of cases and in areas of epidemiological silence. Regional inequalities in the availability of hospital beds, access to health services, and hospitalization criteria may also influence the spatial detection of severe cases. In addition, inconsistencies in the age variable’s completion may have affected the magnitude of the estimates.

The results of the study indicated higher morbidity among children aged 0–4 years and adolescents aged 15–19 years about the impact of COVID-19 on child health. For children under 5 years of age, this vulnerability may be attributed to the immaturity of the immune and respiratory systems, as well as the lack of vaccine eligibility in the first months of life, both of which increase the risk of progression to severe disease [5,9].

Among adolescents, comorbidities, chronic diseases, and behaviors that increase exposure to the virus may be associated with the observed higher incidence. These elements, together with inequalities in access to vaccination and health services, underscore the need for targeted strategies to prevent and control the disease in these age groups [24].

The occurrence of clusters of higher risk in certain periods reflected the interaction between the circulation of more transmissible variants and the still limited availability of protection strategies, especially before the expansion of vaccination to younger age groups. The second wave of the pandemic, which occurred in 2021, showed a significant increase in the number of cases and deaths, which contributed to the formation of high-risk clusters, especially among adolescents aged 15–19 years. This scenario was largely driven by the spread of the Gamma variant in Brazil [25,26].

The third wave of the pandemic, driven by the predominance of the Omicron variant, was characterized by high transmission, a marked increase in cases, and higher mortality in certain population subgroups compared with the second wave [25]. In the Brazilian context, however, the introduction of COVID-19 vaccination from June 2021 onward, with the inclusion of the population aged 12 years or older, may have influenced the impact of this phase of the pandemic among adolescents. From that period onward, the predominant identification of protective clusters for cases and deaths reinforced the interpretation of a progressive process of pandemic control, possibly associated with the expansion of vaccination coverage.

The spatial patterns identified revealed persistent regional inequalities, with a greater concentration of high-risk clusters for deaths in the North and Northeast regions, areas historically marked by greater social vulnerabilities. These inequalities reflect socioeconomic disparities that shape a pattern of vulnerability related to precarious housing, inadequate sanitation, and limited access to health services [26].

The contrast between the high-risk cluster for cases among children aged 0–4 years in the South and Southeast regions and the high-risk cluster for deaths in the North and Northeast regions suggests the influence of structural inequalities in access to and capacity of health services. In the South and Southeast regions, the greater availability of diagnostic services and pediatric hospital care may have favored the detection of severe cases, resulting in higher observed incidence without a proportional increase in mortality [27,28]. In the North and Northeast regions, limitations in timely access to diagnosis and hospital infrastructure may contribute to worse clinical outcomes, as reflected in higher mortality even with fewer detected cases [28].

There is evidence that contexts of greater social inequality are associated with worse COVID-19 outcomes in the pediatric population [28]. Other studies have also aligned with these findings, indicating higher morbidity and mortality in the North and Northeast regions, where children and adolescents are up to three times more likely to die from COVID-19 compared with the South, Southeast, and Central-West regions [26].

The analysis of COVID-19 vaccination coverage revealed marked spatial heterogeneity across Brazilian municipalities and regions, with the persistence of areas with low immunization among children and adolescents. The North region consistently showed the lowest coverage across all age groups, a pattern that may favor the continued circulation of the virus.

This pattern is consistent with national evidence indicating difficulties in expanding child and adolescent vaccination in socially vulnerable territories [8]. Logistical barriers, inequalities in access to health services, and unfavorable socioeconomic conditions, together with misinformation, have been identified as determinants of these disparities [9]. The findings reinforce the need for regionally focused strategies to expand access to and coverage of vaccination in the most vulnerable regions and age groups.

In Brazil, vaccination of children and adolescents is a fundamental right and a duty of the State, guaranteed by the Statute for Children and Adolescents (*Estatuto da Criança e do Adolescent*e, ECA), which establishes the obligation to ensure universal and equitable access to recommended vaccines [29]. Overcoming the inequities observed in vaccination coverage is essential for protecting child health, strengthening the health system response, and reducing inequalities in the response to health emergencies.

This study highlighted important regional inequalities in COVID-19 morbidity and mortality, as well as in vaccination coverage among children and adolescents in Brazil. The findings indicated greater vulnerability in certain contexts, especially in regions historically marked by social inequities and unequal access to health services. By identifying areas and age groups with higher risk and lower levels of vaccination protection, the results provide relevant evidence to support the planning of more equitable and targeted immunization actions, contributing to the strengthening of public policies for child health and to the reduction of inequalities in the response to future health emergencies.

## Data Availability

The database and analysis code used in this research are available at doi.org/10.17605/OSF.IO/T4CMA [30].
