# Supplementary material for: Regional inequalities in vaccination coverage and COVID-19 risk among children and adolescents: spatial analysis, Brazil, 2020–2024
Source: Epidemiol Serv Saude. 2026 Jul 24;35:e20251042. doi: 10.1590/S2237-962220v35e20251042.en (PMC13686397; doi:10.1590/S2237-962220v35e20251042.en)
Supplement: Translation

# Translation: Desigualdades regionais na cobertura vacinal e no risco de covid-19 entre crianças e adolescentes: análise espacial, Brasil, 2020-2024

**DOI:** 10.1590/S2237-962220v35e20251042.pt

**Authors:** Reginaldo Bazon Vaz Tavares, Yan Mathias Alves, Nathalia Zini, Ariela Fehr Tártaro, Maria Eduarda Pagano Pelodan, Luanne Karolyne Leal dos Santos, Daniele de Moraes Pinheiro, Tatiana Ferraz de Araújo Alecrim, Ricardo Alexandre Arcêncio

**Affiliations:** 1 Universidade de São Paulo Escola de Enfermagem de Ribeirão Preto Ribeirão Preto SP Brasil Universidade de São Paulo, Escola de Enfermagem de Ribeirão Preto, Ribeirão Preto, SP, Brasil

**Keywords:** Covid-19, Criança, Vacinas, Risco, Análise Espacial

## Abstract

**Objetivo:**

Identificar aglomerados espaço-temporais de risco para casos e para óbitos por síndrome respiratória aguda grave (SRAG) causada pela covid-19 e caracterizar a distribuição espacial da cobertura vacinal entre crianças e adolescentes de 0-19 anos no Brasil.

**Métodos:**

Estudo de análise espacial com dados secundários dos 5.570 municípios do Brasil. Foram considerados casos e óbitos por SRAG causados pela covid-19 em crianças e adolescentes de 0-19 anos notificados entre março de 2020 e dezembro de 2024 no Sistema de Informação da Vigilância Epidemiológica da Gripe. Foram analisados registros de vacinação entre janeiro de 2021 e dezembro de 2024. As taxas de cobertura vacinal foram calculadas por faixa etária e esquema vacinal. Os aglomerados espaço-temporais foram identificados pela técnica de varredura espacial.

**Resultados:**

Foram registrados 74.470 casos e 3.749 óbitos na população de 0-19 anos. Identificaram-se aglomerados significativos de alto e de baixo risco, inclusive um de proteção para casos entre 0-19 anos (risco relativo [RR] 0,28; intervalos de confiança de 95% [IC95%] 0,27; 0,29) e outro de alto risco para óbitos nas regiões Norte, Nordeste e Centro-Oeste (RR 3,28; IC95% 3,00; 3,59). A vacinação primária alcançou elevada cobertura (85,2%), especialmente entre adolescentes de 15-19 anos (96,8%), e a dose de reforço permaneceu insuficiente (23,1%). A distribuição espacial evidenciou heterogeneidade, com menores coberturas vacinais no Norte e no Centro-Oeste.

**Conclusão:**

Evidenciaram-se desigualdades espaço-temporais nos casos e nos óbitos por SRAG por covid-19 entre crianças e adolescentes, com aglomerados de risco e de heterogeneidade na cobertura vacinal, marcada por alta cobertura primária e por baixo reforço, especialmente no Norte e no Centro-Oeste.

**Aspectos éticos**

Esta pesquisa utilizou bancos de dados de domínio público e anonimizados.

## Introdução

A pandemia de covid-19, causada pelo vírus SARS-CoV-2, impôs grandes desafios aos sistemas de saúde em todo o mundo [1]. O Brasil foi um dos países mais severamente afetados por essa emergência de saúde, com um total de 39.295.043 casos e de 716.509 mortes até agosto de 2025 [2]. Desde o início da pandemia, sabe-se que a doença apresenta maior gravidade em adultos e em idosos, principalmente com comorbidades [3].

Em crianças e em adolescentes a covid-19 tende a ser menos grave, com quadros leves e com menor letalidade que em adultos [3,4,5]. Contudo, hospitalizações, óbitos e complicações como a síndrome inflamatória multissistêmica pediátrica foram frequentes, sobretudo em contextos de maior vulnerabilidades, em países de baixa e de média renda, entre portadores de comorbidades e nos primeiros anos de vida [4].

O Brasil já foi o país com a maior taxa de mortalidade por covid-19 em crianças e em adolescentes, com registro de 23,6 mortes por 1 milhão de crianças em 2020 [4]. Isso evidencia a necessidade de estratégias mais eficazes e equitativas de proteção desse grupo. A inclusão progressiva de crianças e de adolescentes como grupo prioritário nas campanhas de vacinação contra a covid-19, seguida da sua incorporação ao calendário vacinal básico, representou um marco importante no enfrentamento da pandemia, já que ampliou a proteção coletiva e contribuiu para a equidade nas ações de saúde.

As coberturas vacinais contra a covid-19 entre crianças e adolescentes (0-19 anos) permanecem baixas em diversos países, o que ajuda a explicar a sua prioridade global de saúde pública alinhada à Agenda 2030 da Organização das Nações Unidas, especialmente ao objetivo de desenvolvimento sustentável 3 (Saúde e Bem-Estar) [6]. Na Austrália, até setembro de 2022, 51,3% das crianças haviam recebido a primeira dose, e 40,4% a segunda; nos Estados Unidos, até fevereiro de 2023, essas proporções foram de 39,0% e de 32,0%, respectivamente; e no Canadá, até 2023, 67,1% da população menor de 18 anos havia recebido ao menos uma dose da vacina [7,8]. Esses níveis insuficientes evidenciam forte heterogeneidade regional e social, associada a desigualdades socioeconômicas e de acesso aos serviços de saúde [8]. No Brasil, observam-se disparidades marcantes, com maiores coberturas no Sul e no Sudeste e menores no Norte e no Nordeste, relacionadas a fatores sociais, geográficos e logísticos [8].

Embora a covid-19 apresente menor gravidade em crianças e adolescentes comparado a adultos, a mortalidade nessa população ainda é significativa, especialmente em contextos socioeconômicos desfavoráveis [9,10,11,12,13]. Deve-se dizer que as desigualdades na cobertura vacinal podem influenciar padrões geográficos de risco e de mortes evitáveis [3,5]. Porém, passada a emergência da pandemia, não se verifica nenhum estudo que tenha buscado analisar esse fenômeno com uso de análises integradas no Brasil.

Este estudo analisa, sob a perspectiva espaço-temporal, a morbimortalidade e a cobertura vacinal contra a covid-19 em crianças e em adolescentes no Brasil, na busca de preencher lacunas na literatura e subsidiar políticas públicas equitativas e alinhadas aos objetivos de desenvolvimento sustentável. O objetivo do estudo foi identificar aglomerados espaço-temporais de risco para casos e óbitos por síndrome respiratória aguda grave (SRAG) causada pela covid-19 e caracterizar a distribuição espacial da cobertura vacinal contra a covid-19 entre crianças e adolescentes de 0-19 anos no Brasil.

## Métodos

### Delineamento

Trata-se de um estudo de analise espacial, com dados secundários de base populacional, com os 5.570 municípios do Brasil.

### Contexto

O Brasil, o maior país da América Latina, possui uma vasta extensão territorial de 8.509.379,576 km^2^ e uma população estimada em 213.421.037 habitantes. Organizado em 26 estados e o Distrito Federal, o país é dividido em cinco regiões: Norte, Nordeste, Centro-Oeste, Sul e Sudeste [14]. Essa dimensão geográfica reflete uma diversidade de processos sociais, políticos e econômicos, que, por sua vez, perpetuam uma profunda desigualdade regional, especialmente no que se diz respeito à distribuição de renda e ao acesso a serviços essenciais como saúde e educação [15].

### Participantes

Foram incluídos todos os casos e os óbitos notificados de SRAG causada por covid-19 em crianças e em adolescentes de 0-19 anos no Brasil entre março de 2020 e dezembro de 2024. Todos os casos incluídos foram confirmados por teste laboratorial, de acordo com os critérios adotados pelo Ministério da Saúde [16].

Para os dados de vacinação contra a covid-19 foram utilizados os registros de doses aplicadas no período de janeiro de 2021 a dezembro de 2024, com a consideração de todos os tipos de vacinas disponíveis e oferecidas nesse período.

### Fonte de dados e mensuração

Os dados de SRAG por covid-19 foram obtidos do Sistema de Informação da Vigilância Epidemiológica da Gripe (SIVEP-Gripe), sistema implementado em 2000 para a vigilância dos vírus respiratórios em circulação no Brasil [17].

Os dados de vacinação contra a covid-19 foram obtidos pelo portal de dados abertos do Ministério da Saúde, por meio do Departamento de Informação e Informática do Sistema Único de Saúde (DATASUS), extraídos em abril de 2025.

Os dados populacionais de acordo com a faixa etária foram obtidos do censo demográfico de 2022, disponibilizados no portal do Instituto Brasileiro de Geografia e Estatística.

### Variáveis

Para este estudo, foi utilizado o número de casos notificados de SRAG causada por covid-19. Os casos foram filtrados de acordo com a classificação do agente etiológico, com a consideração apenas daqueles confirmados por critério laboratorial que detectaram a presença do SARS-CoV-2. A idade para agregação dos grupos etários foi definida com base no campo “idade” informado em cada notificação. Os registros em que foram identificados valores inconsistentes ou sem preenchimento foram excluídos.

Para contabilizar as mortes por covid-19, utilizou-se a classificação de desfecho dos casos de SRAG por covid-19, com a consideração dos casos cujo encerramento do tratamento foi registrado como óbito e com o seguimento dos mesmos critérios de seleção descritos anteriormente.

Ambos os conjuntos de dados foram estratificados por grupos etários, a saber: 0-4 anos, 5-9 anos, 10-14 anos, 15-19 anos e 0-19 anos. Essa definição foi baseada na padronização de taxas por grupos etários preconizada pela Organização Mundial da Saúde [18] para possibilitar a comparabilidade dos resultados em outros contextos.

As taxas de cobertura vacinal foram calculadas em nível municipal e em nacional, com base nos registros de aplicação das doses da vacina contra a covid-19. Foram consideradas três categorias de cobertura: (i) esquema primário (dose única e segunda dose), (ii) dose de reforço e (iii) terceira dose. A partir disso, as taxas de cobertura foram calculadas conforme fórmula:


$$\mathrm{Cobertura}\;\mathrm{vacinal}=\frac{\mathrm{número}\;\mathrm{de}\;\mathrm{doses}\;\mathrm{aplicadas}\;\;(\mathrm{segundo}\;\mathrm{faixa}\;\mathrm{etária}\;e\;\mathrm{categoria}\;\mathrm{de}\;\mathrm{cobertura})\;}{\mathrm{população}\;\mathrm{por}\;\mathrm{faixa}\;\mathrm{etária}}*100$$

A vacinação contra a covid-19 teve início no Brasil em 17 de janeiro de 2021. A vacinação de crianças e de adolescentes iniciou em 11 de junho de 2021, com a aprovação da vacina da Pfizer para aplicação em adolescentes de 12-17 anos. Em dezembro do mesmo ano, iniciou-se a vacinação para crianças de 5-11 anos com a mesma vacina [19].

Em julho de 2022 crianças de 3-5 anos passaram a poder ser vacinadas com a vacina CoronaVac. Em setembro de 2022, o Ministério da Saúde aprovou a vacina Pfizer Pediátrica para crianças de 6 meses a menores de 5 anos. A partir de novembro de 2022, passou a ser recomendada a vacinação com a vacina bivalente em maiores de 12 anos para pessoas com comorbidades e/ou imunossupressão [19].

Para a análise espacial, foi utilizado o município de residência para determinar o município de ocorrência de cada caso, óbito ou registro vacinal. A data de notificação foi considerada como referência temporal das ocorrências de casos e de óbitos, e a data de aplicação da vacina para registro vacinal.

### Métodos de análise estatística Detecção de aglomerados

Para a detecção de aglomerados espaço-temporais de ocorrência de casos e de óbitos por covid-19 em crianças e em adolescentes foi utilizada a técnica de Scan Statistics [20,21], com utilização dos 5.570 municípios brasileiros como unidade de análise. Nessa técnica, a informação sobre a ocorrência do evento é associada a um único ponto no mapa, definido para cada unidade de análise estudada, denominado centroide. A partir disso, toda a área de estudo é examinada por meio da criação de cilindros de dimensões variáveis em torno dos centroides.

A identificação de aglomerados ocorre por meio do cálculo do número de eventos esperados e observados. Quando o número de eventos esperados difere significativamente do número de eventos observados (seja maior ou menor), define-se um aglomerado. Caso essa diferença não seja significativa, a base (dimensão espacial) ou a altura (dimensão temporal) do cilindro é ampliada, até que todos os centroides e todo o período do estudo sejam testados [22,23].

O teste estatístico considera como hipótese nula (H₀) a ausência de aglomerados na região do estudo, e como hipótese alternativa (H₁) que uma determinada região Z constitua um aglomerado. Para os aglomerados identificados, calcula-se o risco relativo (RR), que determina se o aglomerado representa uma área de risco (>1) ou de proteção (<1) [22,23].

Adotou-se uma abordagem retrospectiva e, por se tratar de dados de contagem (número de casos e de óbitos), utilizou-se o modelo de Poisson. Os parâmetros da análise incluíram: não sobreposição geográfica dos aglomerados; aglomerados com tamanho máximo de 50% da população exposta e 50% do período do estudo; formato circular dos aglomerados; 999 replicações de Monte Carlo; e precisão temporal em meses, no período de março de 2020 a dezembro de 2024. Foram considerados estatisticamente significativos os aglomerados com p-valor<0,05. A análise foi realizada no software SaTScan 10.0.2 (https://www.satscan.org/).

Para a visualização espacial dos resultados, foram construídos mapas coropléticos que continham os aglomerados identificados e os seus respectivos valores de RR e de intervalos de confiança de 95% (IC95%), com a utilizando o software ArcGIS 10.5 (https://www.esri.com/). Também foram apresentados os valores observados e esperados de casos e de óbitos para cada aglomerado.

### Séries temporais

As séries temporais mensais dos casos e dos óbitos foram elaboradas para cada faixa etária, com a consideração da ocorrência de eventos dentro dos aglomerados encontrados. As análises foram conduzidas com utilização da linguagem R, através do software R Studio (versão 2025.05.1).

### Distribuição espacial da cobertura vacinal

Para mapear a cobertura vacinal contra a covid-19, foram construídos mapas coropléticos utilizando os municípios como unidade de análise, estratificados por faixa etária e por tipo de esquema vacinal. Os mapas foram elaborados na linguagem R, por meio do software RStudio (versão 2025.05.1).

## Resultados

Foram registrados um total de 74.470 casos de SRAG causada por covid-19 e 3.749 óbitos entre crianças e adolescentes de 0-19 anos no período de março de 2020 a dezembro de 2024.

A Tabela 1 apresentou as taxas anuais de incidência e de mortalidade por SRAG segundo faixa etária. Observou-se maior incidência entre crianças de 0-4 anos, com 71,7 casos por 100 mil habitantes, seguida pelos adolescentes de 15-19 anos (17,7 casos/100 mil) e pelas crianças de 5-9 anos (15,9 casos/100 mil).

**Tabela 1:** Taxas de incidência e de mortalidade por síndrome respiratória aguda grave causada por covid-19 entre crianças e adolescentes de 0-19 anos, segundo faixa etária. Brasil, 2020-2024 (n=74.470)

Faixa etária (anos)	Taxa de incidência anual (casos/100 mil habitantes)	Taxa de mortalidade anual (mortes/100 mil habitantes)
0-19	27,3	1,4
0-4	69,4	3,0
5-9	15,4	0,5
10-14	10,8	0,6
15-19	17,1	1,5

As maiores taxas de mortalidade também foram registradas no grupo de 0-4 anos, com 3,1 óbitos por 100 mil habitantes, seguido pelos adolescentes de 15-19 anos (1,5 óbitos/100 mil). As menores taxas de mortalidade foram observadas entre crianças de 5-9 anos (0,5 óbitos/100 mil) e no grupo de 10-14 anos (0,6 óbitos/100 mil).

A análise de varredura espaço-temporal identificou aglomerados estatisticamente significativos de risco elevado e de proteção de casos (Figura 1) e óbitos (Figura 2), com a consideração de todos os grupos etários estudados.

**Figura 1:** Aglomerados espaço-temporais e séries temporais de casos notificados de síndrome respiratória aguda grave causada por covid-19 em crianças e em adolescentes de (A) 0-19 anos, (B) 0-4 anos, (C) 5-9 anos, (D) 10-14 anos e (E) 15-19 anos. Brasil, 2020-2024 (n=74.470)

**Figura 2:** Aglomerados espaço-temporais e séries temporais de óbitos notificados de síndrome respiratória aguda grave causada por covid-19 em crianças e em adolescentes de (A) 0-19 anos, (B) 0-4 anos, (C) 5-9 anos, (D) 10-14 anos e (E) 15-19 anos. Brasil, 2020-2024 (n=3.749)

Na faixa etária 0-19 anos (Figura 1A), foi detectado um aglomerado com RR 0,28 (IC95% 0,27; 0,29) composto por uma população de 27.242.116 pessoas, com 6.444 casos observados e 18.592,36 casos esperados, com abrangência principalmente nas regiões Norte, Nordeste e Centro-Oeste.

Na faixa etária 0-4 anos (Figura 1B), foram identificados dois aglomerados. O aglomerado 1 teve RR 3,42 (IC95% 3,27; 3,57), composto por uma população de 6.334.196 pessoas, por 7.885 casos observados e 2.637,79 esperados, com abrangência principalmente nas regiões Sul e em partes do Centro-Oeste e do Sudeste. O aglomerado 2 teve RR 0,31 (IC95% 0,30; 0,32), composto por uma população de 4.509.684 pessoas, por 2.800 casos observados e 7.830,88 esperados, com abrangência principalmente nas regiões Nordeste e em partes das regiões Sudeste e Norte.

Na faixa etária 5-9 anos (Figura 1C), foi identificado um aglomerado com RR 0,27 (IC95% 0,25; 0,29), composto por uma população de 6.869.811 pessoas, por 870 casos observados e 2.641,86 esperados, com abrangência em toda a região Nordeste e em partes das regiões Centro-Oeste, Sudeste e Norte.

Na faixa etária 10-14 anos (Figura 1D), foi identificado um aglomerado com RR 3,23 (IC95% 3,05; 3,42), composto por uma de população de 6.660.105 pessoas, com 3.596 casos observados e 1.675,66 esperados, com abrangência nas regiões Sul, Sudeste e Centro-Oeste.

Na faixa etária 15-19 anos (Figura 1E), foi identificado um aglomerado com RR 4,36 (IC95% 4,16; 4,57), composto por uma população de 7.174.512 pessoas, com 6.195 casos observados e 214,05 casos esperados, com abrangência em toda a região Centro-Oeste e em partes das regiões Norte, Sudeste e Norte.

Em relação aos óbitos por covid-19, foi detectado um aglomerado com RR 3,28 (IC95% 3,00; 3,59) na faixa etária 0-19 anos (Figura 2A), composto por uma população de 25.563.551 pessoas, com 1.602 óbitos observados e 694,45 casos esperados, com abrangência nas regiões Norte, Nordeste e Centro-Oeste.

Na faixa etária 0-4 anos (Figura 2B), foi identificado um aglomerado com RR 3,47 (IC95% 3,07; 3,93), composto por uma população de 5.975.208 pessoas, 846 óbitos observados e 356,09 esperados, com abrangência nas regiões Norte, Nordeste e Centro-Oeste.

Na faixa etária 5-9 anos (Figura 2C), foi identificado um aglomerado com RR 0,11 (IC95% 0,06; 0,21), composto por uma população de 6.874.405 pessoas, com 10 óbitos observados e 75,5 esperados, com abrangência nas regiões Norte, Nordeste e Centro-Oeste.

Foi identificado um aglomerado, na faixa etária 10-14 anos (Figura 2D), com RR 0,04 (IC95% 0,01; 0,11), composto por uma de população de 6.607.633 pessoas, com 4 óbitos observados e 83,76 esperados, com abrangência em toda a região Norte, Nordeste e Centro-Oeste.

Na faixa etária 15-19 anos (Figura 2E), foi identificado um aglomerado com RR 0,05 (IC95% 0,03; 0,09), composto por uma população de 7.117.967 pessoas, com 14 óbitos observados e 214,05 casos esperados, com abrangência principalmente na região Sudeste e em partes das regiões Norte, Nordeste e Centro-Oeste.

A Tabela 2 mostrou alta cobertura do esquema primário contra covid-19 entre crianças e adolescentes, sobretudo de 15-19 anos (96,8%). Já a adesão ao reforço foi heterogênea, com variação de 0,4% (0-4 anos) a 49,7% (15-19 anos). A terceira dose apresentou baixa cobertura em todas as faixas etárias, o que indicou desafios na ampliação vacinal.

**Tabela 2:** Cobertura vacinal da população de 0-19 anos no Brasil, segundo faixa etária e esquema de vacinação contra a covid-19. Brasil, 2020-2024 (n=120.607.559)

Esquema vacinal/faixa etária (anos)	Doses totais	População	Cobertura (%)
**Vacinação primária**			
0-19	46.421.022	54.505.203	85,2
0-4	7.964.519	12.704.860	62,7
5-9	11.575.968	13.749.440	84,2
10-14	11.575.968	13.749.440	84,2
15-19	13.918.636	14.375.942	96,9
**Dose de reforço**			
0-19	12.572.858	54.505.203	23,1
0-4	45.484	12.704.860	0,4
5-9	1.465.249	13.749.440	10,7
10-14	3.915.185	13.674.961	28,6
15-19	7.146.940	14.375.942	49,7
**Terceira dose**			
0-19	2.002.865	54.505.203	3,7
0-4	1.062.030	12.704.860	8,4
5-9	440.765	13.749.440	3,2
10-14	322.401	13.674.961	2,4
15-19	177.669	14.375.942	1,2

A Figura 3 apresentou a distribuição espacial da cobertura vacinal contra a covid-19 entre crianças e adolescentes no Brasil, por grupos etários e por tipo de esquema vacinal (primário, primeira dose de reforço e terceira dose de reforço).

**Figura 3:** Distribuição espacial da taxa de cobertura vacinal contra covid-19 entre crianças e adolescentes, por faixa etária, com esquema primário: (A) 0-19 anos, (D) 0-4 anos, (G) 5-9 anos, (J) 10-14 anos, (M) 15-19 anos; com dose de reforço: (B) 0-19 anos, (E) 0-4 anos, (H) 5-9 anos, (K) 10-14 anos, (N) 15-19 anos); e com terceira dose: (C) 0-19 anos, (F) 0-4 anos, (I) 5-9 anos, (L) 10-14 anos, (O) 15-19 anos). Brasil, 2020-2024 (n=120.607.559)

Houve alta cobertura do esquema primário, sobretudo a partir de 5 anos, com maiores percentuais nas regiões Sul, Sudeste e Nordeste (Figuras 3G, 3J, 3M). Já o Norte e o Centro-Oeste apresentaram bolsões de baixa cobertura, principalmente entre menores de 4 anos (Figura 3D). A cobertura da dose de reforço (Figura 3E) foi mais heterogênea, com baixas coberturas nas faixas mais jovens, enquanto a terceira dose mostrou baixa adesão em todas as idades (Figuras 3F, 3I, 3L, 3O), com forte desigualdade territorial e menor cobertura no Centro-Oeste.

## Discussão

Os achados indicaram que a ocorrência de casos e de óbitos por covid-19 em crianças e em adolescentes no Brasil apresentou marcada heterogeneidade territorial. A identificação de áreas de maior risco e de proteção evidencia que a distribuição da doença não foi homogênea entre municípios e regiões, o que refletiu a influência de contextos epidemiológicos e sociais distintos. Esses resultados reforçaram a relevância da abordagem espaço-temporal para compreender a dinâmica da covid-19 nesse grupo etário e para subsidiar estratégias de vigilância e de intervenção em territórios específicos.

Este estudo apresentou algumas limitações. O uso do SIVEP-Gripe restringiu a análise aos casos graves de covid-19, com a não captação de infecções leves ou assintomáticas, mais frequentes em crianças e em adolescentes. Deve-se considerar o efeito da subnotificação e do viés de notificação, especialmente em áreas socialmente vulneráveis, o que pode resultar na subestimação de casos e em áreas de silenciamento epidemiológico. Desigualdades regionais na oferta de leitos, no acesso aos serviços de saúde e nos critérios de internação também podem influenciar a detecção espacial dos casos graves. E inconsistências no preenchimento da variável idade podem ter afetado a magnitude das estimativas.

Os resultados do estudo apontaram maior morbidade em crianças de 0-4 anos e em adolescentes de 15-19 anos no que se refere ao impacto da covid-19 na saúde infantil. Para os menores de 5 anos, essa vulnerabilidade pode ser atribuída à imaturidade do sistema imunológico e respiratório, além da impossibilidade de vacinação nos primeiros meses de vida, fatores que aumentam o risco de evolução para quadros graves [5,9]. 

Entre adolescentes, fatores como presença de comorbidades, doenças crônicas e comportamentos que favorecem maior exposição ao vírus podem estar relacionados à maior incidência observada. Esses elementos, associados a desigualdades no acesso à vacinação e a serviços de saúde, reforçam a necessidade de estratégias direcionadas para a prevenção e o controle da doença nesses grupos etários [24].

A ocorrência de aglomerados de maior risco em determinados períodos refletiu a interação entre a circulação de variantes mais transmissíveis e a disponibilidade ainda limitada de estratégias de proteção, especialmente antes da ampliação da vacinação para faixas etárias mais jovens. A segunda onda da pandemia, ocorrida em 2021, apresentou aumento significativo no número de casos e de óbitos, o que contribuiu para a formação de aglomerados de alto risco, especialmente entre adolescentes de 15-19 anos. Esse cenário foi impulsionado, em grande parte, pela disseminação da variante gama no Brasil [25,26].

A terceira onda da pandemia, associada à predominância da variante ômicron, caracterizou-se por elevada transmissibilidade e expressivo aumento no número de casos, além de maior mortalidade em determinados subgrupos populacionais quando comparada à segunda onda [25]. No contexto brasileiro, contudo, a introdução da vacinação contra a covid-19 a partir de junho de 2021 com inclusão da população com 12 anos ou mais pode ter influenciado o impacto dessa fase da pandemia entre adolescentes. A partir desse período, a identificação predominante de aglomerados de proteção para casos e óbitos reforçou a interpretação de um processo progressivo de controle da pandemia, possivelmente associado à ampliação da cobertura vacinal.

Os padrões espaciais identificados evidenciaram desigualdades regionais persistentes, com maior concentração de aglomerados de alto risco de óbitos nas regiões Norte e Nordeste, áreas historicamente marcadas por maiores vulnerabilidades sociais. Essas desigualdades refletiram disparidades socioeconômicas que configuram um padrão de vulnerabilidade relacionado a habitação precária, saneamento deficitário e acesso limitado a serviços de saúde [26].

O contraste entre o aglomerado de alto risco para casos em crianças de 0-4 anos nas regiões Sul e Sudeste e o aglomerado de alto risco para óbitos nas regiões Norte e Nordeste sugeriu a influência de desigualdades estruturais no acesso e na capacidade dos serviços de saúde. Nas regiões Sul e Sudeste, a maior disponibilidade de diagnóstico e de cuidados hospitalares pediátricos pode ter favorecido a detecção de casos graves, o que resultou em maior incidência observada sem aumento proporcional da mortalidade [27,28]. Já nas regiões Norte e Nordeste, limitações no acesso oportuno ao diagnóstico e a infraestrutura hospitalar podem contribuir para piores desfechos clínicos, o que se reflete em maior mortalidade mesmo diante de menor número de casos detectados [28].

Existem evidências de que contextos de maior desigualdade social estão associados a piores desfechos da covid-19 na população pediátrica [28]. Outros estudos também se alinharam aos achados, com apontamento de maior morbimortalidade nas regiões Norte e Nordeste, onde crianças e adolescentes têm até três vezes mais chances de morrer por covid-19 em comparação às regiões Sul, Sudeste e Centro-Oeste [26].

A análise da cobertura vacinal contra a covid-19 revelou marcada heterogeneidade espacial entre municípios e regiões do Brasil, com a persistência de áreas de baixa imunização entre crianças e adolescentes. Destacou-se a região Norte, que apresentou consistentemente as menores coberturas em todos os grupos etários, cenário que pode favorecer a manutenção da circulação viral.

Esse padrão é coerente com evidências nacionais que indicaram dificuldades na ampliação da vacinação pediátrica em territórios socialmente vulneráveis [8]. Barreiras logísticas, desigualdades de acesso aos serviços de saúde e fatores socioeconômicos desfavoráveis, associados à desinformação, têm sido apontados como determinantes dessas disparidades [9]. Os achados reforçaram a necessidade de estratégias territorialmente orientadas para ampliar o acesso e a adesão à vacinação nas regiões e nos grupos etários mais vulneráveis.

No Brasil, a vacinação de crianças e de adolescentes configura-se como um direito fundamental e um dever do Estado, assegurado pelo Estatuto da Criança e do Adolescente, que estabelece a obrigação de garantir acesso universal e equitativo às vacinas recomendadas [29]. A superação das iniquidades observadas na cobertura vacinal é condição essencial para a proteção da saúde infantil, o fortalecimento da resposta do sistema de saúde e a redução das desigualdades no enfrentamento de emergências sanitárias.

Este estudo evidenciou importantes desigualdades regionais na morbimortalidade por covid-19 e na cobertura vacinal entre crianças e adolescentes no Brasil. Os achados indicaram maior vulnerabilidade em determinados contextos, especialmente em regiões historicamente marcadas por iniquidades sociais e de acesso aos serviços de saúde. Ao identificar áreas e grupos etários com maior risco e menores níveis de proteção vacinal, os resultados ofereceram subsídios relevantes para o planejamento de ações de imunização mais equitativas e direcionadas, o que contribui para o fortalecimento das políticas públicas de saúde infantil e para a redução de desigualdades no enfrentamento de emergências sanitárias futuras.

## Data Availability

O banco de dados e os códigos de análise utilizados na pesquisa estão disponíveis em doi.org/10.17605/OSF.IO/T4CMA [30].
